# Targeting the bacterial stringent response to combat human pathogens

**DOI:** 10.3389/fimmu.2025.1663548

**Published:** 2025-09-01

**Authors:** Filip Gąsior, Katarzyna Bryszkowska, Wiktoria Klasa, Katarzyna Potrykus

**Affiliations:** Department of Bacterial Molecular Genetics, Faculty of Biology, University of Gdańsk, Gdańsk, Poland

**Keywords:** stringent response, bacterial stress response, alarmone, (p)ppGpp, host-pathogen interaction, RelA/SpoT homologs (RSH)

## Abstract

In the era of increasing bacterial antibiotic resistance, finding new ways of combating pathogens is especially important. An attractive possibility is targeting bacterial survival strategies that microorganisms employ either to evade the host immune-responses or to adapt to the hostile environment encountered once inside the host. An example of the latter is the stringent response, mediated by guanosine penta- and tetra-phosphate, collectively referred to as (p)ppGpp. These molecules (alarmones) are responsible for switching bacterial gene expression and metabolism to allow survival under various stresses, such as nutritional deprivation and oxidative stress. (p)ppGpp turnover is mediated by various enzymes belonging to the RSH (RelA-SpoT homolog) family, some of which are capable of both, (p)ppGpp synthesis and hydrolysis, while others can perform only one of these functions. In this minireview, we discuss strategies that aim to disrupt or modulate the stringent response either by inhibiting these enzymes or on the contrary – enhancing their activities, as that goal can be achieved by several ways, i.e. blocking (p)ppGpp synthesis, inducing its synthesis or blocking its hydrolysis.

## 1 Introduction

The process of colonization of the host organism by pathogenic bacteria is a complex and multi-stage phenomenon. To effectively colonize the host, pathogens must overcome numerous protective barriers, including physical structures, chemical factors and highly specialized mechanisms of the immune response. In addition, the most common obstacles include nutrient deficiency - the host limits, e.g. amino acids (1), carbon availability (2) and essential metal ions (e.g. iron) (3). Other challenges that pathogens have to face is oxidative (4) and nitrosative stress (5). In some locations (e.g. the digestive or urinary tract), bacteria must also adapt to changing osmotic conditions, rapid pH fluctuations and the action of lytic enzymes and antimicrobial peptides (6–8). In response, bacteria have evolved a variety of adaptive strategies that allow them to successfully survive and proliferate in the hostile host environment. Many of these strategies are controlled by the stringent response, mediated by the guanosine penta- and tetra-phosphate, collectively referred to as (p)ppGpp, which alert the cell to a multitude of stress factors – including nutrient starvation (i.e. amino acid, carbon, nitrogen, phosphate, iron and lipid limitation) and physico-chemical stresses (such as osmotic, oxidative, and acid stress) (9, 10). Synthesis of (p)ppGpp is carried out from GTP (in case of pppGpp) or GDP (ppGpp) and ATP which is the donor of the pyrophosphate group (transferred from ATP to 3′ position of GTP or GDP) (9).

At its core, the stringent response aims to inhibit growth and activate survival mechanisms at the same time. In particular, this response has been reported to be involved at various stages of bacterial infection, such as adherence, invasion, immune evasion, bacterial cell dissemination, biofilm formation, sporulation, persistence and antibiotic tolerance, including but not limited to production of specific enzymes (e.g. catalases that protect pathogens from oxidative stress) and structures (such as fimbriae), as well as activation of pathogenicity-related gene expression (10, 11). Although (p)ppGpp affects a multitude of pathways, the impact on bacterial virulence is typically regulated through transcriptional changes – either by its direct binding to the RNA polymerase (e.g. in proteobacteria) or by causing a decrease in GTP levels (as in Bacillota, Actinobacteria, and Deinococcus-Thermus) (10, 12, 13).

The (p)ppGpp turnover is mediated by various enzymes, most notably those belonging to the RSH (RelA-SpoT homolog) superfamily. Many of these enzymes possess both, the (p)ppGpp synthetase and hydrolase domains along with regulatory domains (the so-called long RSH enzymes), while others possess short catalytic domains only (i.e. small alarmone synthetases (SASs) and small alarmone hydrolases (SAHs)) (14). In addition to bifunctional long RSH enzymes (e.g. Rel in *Bacillus subtilis* or SpoT in *Escherichia coli*) present in almost all bacteria, most β- and γ-proteobacteria also possess a synthetase-only long RSH (e.g. RelA in *E. coli*) (13, 15). In that case, each long RSH enzyme can respond to a different environmental stress, e.g. *E. coli* RelA responds to amino acid starvation while *E. coli* SpoT responds to all other stresses (9). In contrast to examples described above, bacteria from the Planctomycetes, Verrucomicrobia, and Chlamydiae phyla (the PVC superphylum) do not possess a long RSH enzyme at all (15).

It should be noted, that the long and short RSH enzymes can coexist in various combinations, for example in addition to Rel, *B. subtilis* contains two SASs – SAS1 (also known as RelQ) and SAS2 (RelP) (16). On the other hand, while all mycobacteria encode a bifunctional long RSH protein (e.g. RelMtb in *Mycobacterium tuberculosis* and RelMsm in *Mycolicibacterium smegmatis*), *M. smegmatis* also possesses a SAS protein – RelZ (which is unusual among SASs as it features an additional RNase HII domain), while *M. tuberculosis* encodes its non-functional ortholog – Rv1366 (17).

RSH enzymes are subject to diverse regulatory mechanisms, including transcriptional control; ligand-mediated regulation through substrates, products, or atypical ligands such as ssRNA; heterologous protein interactions; as well as indirect regulation via crosstalk with other secondary messenger nucleotides (18). Moreover, oligomerization may play an important role in regulation of RSH enzymes activity - in *E. coli*, RelA forms dimers via C-terminal domain (CTD) interactions that lower its synthetase activity, while RelMtb forms less active trimers that dissociate upon substrate or product binding. However, this seems not to be limited to long RSH enzymes, as even though lacking the CTD, activity of SAS1 from *B. subtilis* is dependent on tetramerization (18).

In addition to the enzymes described above, there is a number of (p)ppGpp metabolizing enzymes that do not belong to the RSH superfamily, such as GppA phosphatase found in γ- and some δ-proteobacteria (19) or NuDiX hydrolases, e.g. NahA from *B. subtilis* or MutT from *E. coli*, that can cleave (p)ppGpp into such derivatives as pGpp or pGp, respectively (10, 20, 21).

## 2 Approaches aimed at targeting the stringent response

### 2.1 (p)ppGpp synthesis and hydrolysis pathways as direct molecular targets in combating different bacteria

**Figure 1:**
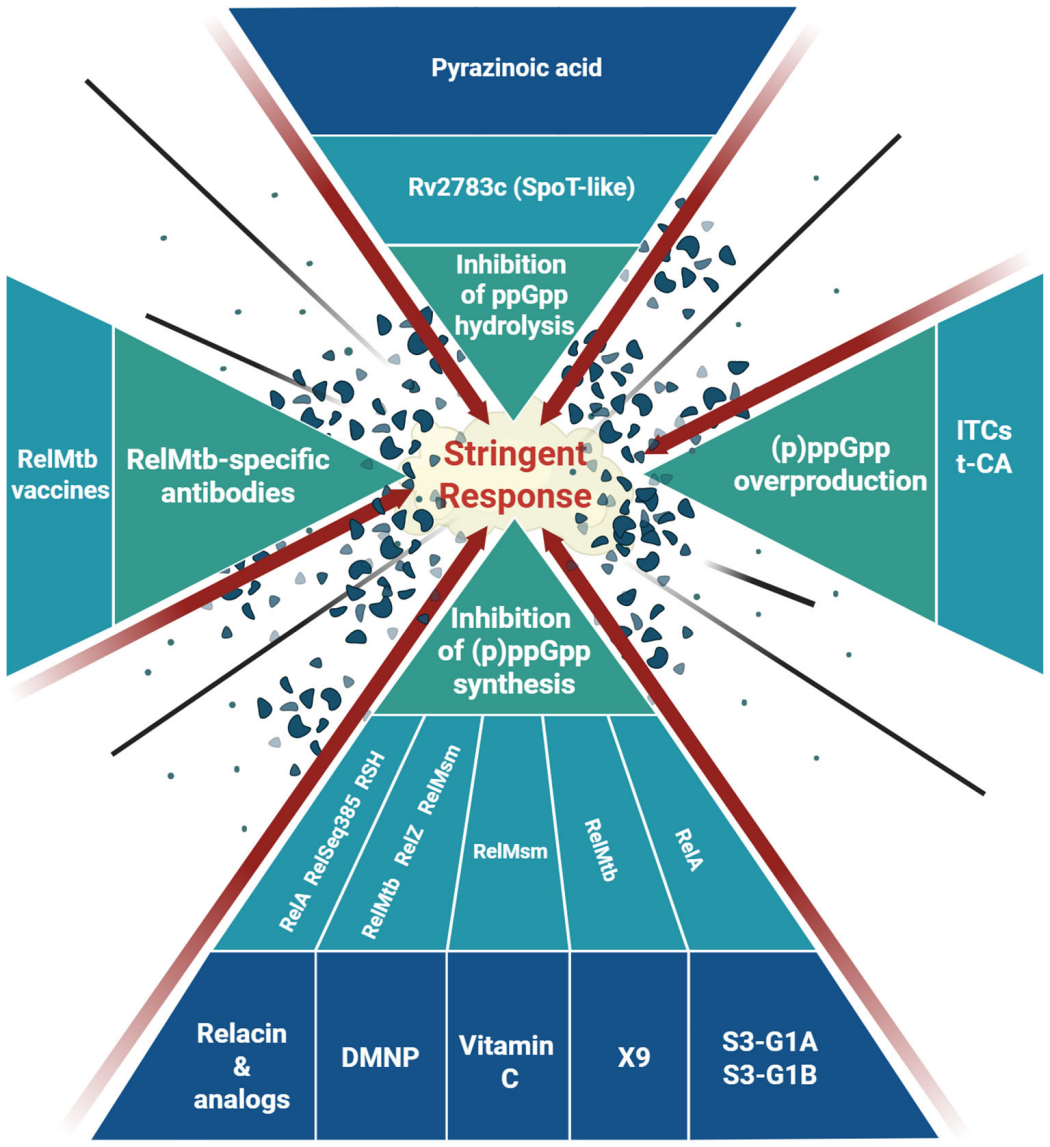
Summary of the current approaches applied to target the bacterial stringent response. See text for details. *Created in BioRender. Gąsior, F. (2025)* *https://BioRender.com/1f62hnc* .

Since (p)ppGpp is the mediator of the stringent response and is required for turning on the survival strategies upon various stresses, including nutrient starvation, as well as for activation of pathogenicity-related gene expression, the most straightforward approach to combat these effects seems to be limitation of (p)ppGpp production. Indeed, most of the antimicrobial strategies based on altering the stringent response focus on that aspect of (p)ppGpp metabolism (**Figure 1**). In that case, (p)ppGpp synthetases are generally the target.

In the first report exploring this idea, several ppGpp analogues were tested, and the one containing bisphosphonate groups at the 5′ and 3′ positions instead of the natural pyrophosphates displayed the most inhibitory effects (22). *In vitro* tests had demonstrated it to cause partial inhibition of RelA from *E. coli* and truncated RelSeq enzyme from *Streptococcus dysgalactiae* subsp. *equisimilis* (RelSeq_1-385_). This analogue most likely competes with GDP and GTP for binding at the active site of these enzymes. However, the use of this compound was not tested *in vivo* (22).

In a subsequent study, another ppGpp analogue, called relacin, was found to be much more efficient at inhibiting (p)ppGpp synthetases than the previous versions. Two synthetases were tested *in vitro*, representing Gram(-) (*E. coli* RelA) and Gram(+) (*Deinococcus radiodurans* RSH) species. Relacin was obtained by replacing ppGpp’s phosphate groups by glycyl-glycine dipeptides and adding an isobutyryl group at the C - 2 position of the guanine base; similarly to the compound used in the previous study, it was designed to bind to the tested synthetases’ active site (23). As with the other ppGpp analogues, relacin had no effect on *E. coli* cells, however, a promising *in vivo* effect was observed for Gram(+) species (*B. subtilis*, *B. anthracis*, *D. radiodurans* and Group A *Streptococcus*) where it was shown to limit (p)ppGpp production (tested in *B. subtilis*), impede entrance into stationary phase (all four species), inhibit biofilm formation (*B. subtilis*) and sporulation (*B. subtilis* and *B. anthracis*). The lack of effect on *E. coli* cells is thought to be due to relacin’s inability to cross the Gram(-) bacteria’s membranes and enter the cell (23). In addition to these studies, it was shown that relacin inhibited RSHCd (RSH enzyme from *Clostridioides difficile*) activity *in vitro*, but was efficient *in vivo* against the epidemic strain of *C. difficile* only in combination with metronidazole or clindamycin (24).

Most other studies that followed the ones described above and which focused on targeting (p)ppGpp synthetases were concerned almost exclusively with mycobacteria. For example, ppGpp analogues created by replacing the ppGpp’s 5′ and 3′ phosphate groups by acetyl groups, adding the acetyl group at the 2′ ribose and adding either the acetyl or isobutyryl group at the C - 2 position of the guanine base (the AC and AB compounds, respectively) were shown to be effective at inhibiting RelMsm (*M. smegmatis* Rel) *in vitro*, with AB being twice as efficient (25). In addition, both compounds reduced (p)ppGpp level and limited bacterial survival under stress conditions (*M. smegmatis*), as well as reduced biofilm formation by *M. smegmatis* and *M. tuberculosis* (25). Importantly, MTT tests on a human lung cancer cell line (H460) and hemolysis tests showed no cytotoxicity and no damage to erythrocyte membranes, respectively, suggesting these compounds could be used in treating human infections (25).

Other studies on targeting the stringent response in mycobacteria were conducted using a truncated RelMtb protein (RelMtb_53-446_), which contains the (p)ppGpp synthetase and hydrolase domains. Two million compounds from the GlaxoSmithKline (GSK) compound library were screened, among which compound X9 showed a strong *in vitro* activity against RelMtb and was effective *in vivo* against *M. tuberculosis* wild type strains, while it had no effect on the Δ*relMtb* mutant strain. X9 also enhanced the activity of isoniazid (INH) which is a standard drug used against mycobacteria (26).

Another example of a successful library screen was reported by (27) where 4 million commercially available compounds from the University of California, San Francisco (UCSF) ZINC database were first screened *in silico* by molecular docking to *E. coli* RelA. Two of them, S3-G1A and S3-G1B, exhibited better parameters than relacin and were shown to be effective in RelA inhibition *in vitro* and *in vivo* (27).

Natural compounds or their synthetic analogues, unrelated to (p)ppGpp structure, were also tested as (p)ppGpp synthetase inhibitors. An intriguing example is the well-known Vitamin C, which was shown to bind to RelMsm (28). *In vivo*, this natural compound prevents biofilm formation by mycobacteria which may be related to its ability to lower cellular (p)ppGpp levels (28). On the other hand, DMNP (4-(4,7-dimethyl-1,2,3,4-tetrahydronaphthalen-1-yl)pentanoic acid) – a synthetic analogue of naturally occurring erogorgiaene (a metabolite found in soft coral *Antillogorgia elisabethae*), was shown to reduce the (p)ppGpp synthetase activity of mycobacterial RelMsm and RelZ (29, 30), as well as RelMtb (31).

An interesting molecular target related to the stringent response is the *M. tuberculosis* Rv2783c protein, which resembles *E. coli* SpoT in its function, but mainly acts as a ppGpp hydrolase. It has been shown that pyrazinoic acid (POA), which is a metabolite of the *M. tuberculosis* directed drug - pyrazinamide (PZA), binds to Rv2783c and blocks ppGpp hydrolysis. This suggests that the effectiveness of PZA against mycobacteria relies on disruption of the stringent response metabolism (32). So far, this is the only promising example of an attempt to directly target (p)ppGpp hydrolysis and not synthesis.

Initially promising results were also reported for peptide 1018 (IDR (innate defense regulator) -1018) which was thought to inhibit the development of biofilm produced by *Pseudomonas aeruginosa* by promoting faster ppGpp degradation (33, 34). However, this was later challenged by a report which showed that a similar inhibitory effect by 1018 was observed for *P. aeruginosa* unable to produce ppGpp (35).

### 2.2 Indirect modulators of the stringent response - a promising strategy against bacterial pathogens

Over the past decade, various reports have also focused on the activity of natural compounds that can indirectly modulate bacterial stringent response by inducing specific cellular stresses. Isothiocyanates (ITC), a group of natural antimicrobial compounds derived from the *Brassicaceae* plant family, was demonstrated to directly induce the stringent response. All ITCs tested were shown to increase (p)ppGpp cellular level to varying degree, with phenethyl ITC being the most active (36). This process was dependent on the presence of *relA*, which suggested induction of the amino acid deprivation-dependent pathway of the stringent response. In enterohemorrhagic *E. coli* (EHEC) ITC led to bacterial growth inhibition and suppression of the Shiga toxin gene expression, whose activation is associated with lambdoid prophage induction (36, 37). This finding is especially important because use of the standard antibiotics alone is discouraged in the treatment of EHEC infections, since here inhibition of bacterial growth is usually accompanied by Shiga toxin production, which can lead to serious health complications (38).

The antibacterial potential of ITC has been also demonstrated against another bacterial pathogen, i.e. *Vibrio cholerae*. Two well-studied ITCs, sulforaphane and phenethyl isothiocyanate, were shown to exert their antibacterial potential via the same route as for EHEC – by the induction of the stringent response (39). Studies conducted on *V. cholerae* demonstrated inhibition of biofilm formation and bacterial growth, as well as a reduction in toxin production by ITCs (39). Interestingly, although ITCs inhibit growth of various bacterial pathogens, their mechanism of action not always involves the stringent response – e.g. in *B. subtilis* the ITC treatment has not led to (p)ppGpp accumulation and its antibacterial effect is due to bacterial membrane integrity disruption (37).

On the other hand, recent research on t-cinnamaldehyde (t-CA) revealed that it disrupts pyruvate metabolism and limits lysine biosynthesis, which in turn leads to metabolic stress that induces *E. coli* RelA activity and consequently indirectly stimulates (p)ppGpp production. This results in EHEC growth inhibition and decreased Shiga toxin production which in turn reduces bacterial toxicity, as observed *in vivo* with the use of the *Galleria mellonella* model (40). Moreover, combining this natural compound with azithromycin enhances its antimicrobial effect against EHEC, indicating potential optimization strategies for antimicrobial therapy (41).

### 2.3 Vaccines against stringent response related proteins

One of the latest approaches in combating pathogenic bacteria by disrupting the stringent response involves the use of DNA vaccines, as exemplified by a vaccine containing four stringent response-related genes from *M. tuberculosis*—*relMtb*, *sigE*, *ppk2*, and *ppx*. The *sigE*, *ppk2* and *ppx* gene products were implicated earlier in the signaling network involving RelMtb (42). Tested in mouse models, this intramuscularly introduced vaccine induced production of RelMtb specific IgG antibodies and activation of CD4+ T cells producing IFN-γ and TNF-α (43). Further studies had shown that exposure of *M. tuberculosis*-infected macrophages to isoniazid (a typically employed mycobacterial drug) strongly upregulated *relMtb* expression, which led to the development of a DNA vaccine targeting this gene-product alone (44). In addition, this vaccine increased bacterial susceptibility to isoniazid and significantly limited *M. tuberculosis* replication after the end of antibiotic treatment (44), thus confirming the choice of RelMtb as an appropriate immunological target to support effective therapy.

## 3 Discussion

Although the approaches described above and summarized in **Figure 1** seem promising and the first molecule specifically inhibiting (p)ppGpp synthesis had been obtained over a decade ago, to the best of our knowledge none of the compounds described above have so far reached the clinical trial phase focused exclusively on targeting the stringent response. However, new light can be shed on already known compounds, e.g. thanks to extensive *in silico* analysis. Recently, a known antituberculosis peptide drug (Pantocin wh-1) was shown by molecular docking to bind to the *Staphylococcus aureus* RelP which may be a promising approach in targeting methicillin-resistant staphylococcal strains (45).

On the other hand, although novel compounds targeting the bacterial stringent response are continually being developed, the high concentrations that are required to demonstrate their activity *in vitro* present a substantial challenge. This is illustrated by the high half-maximal inhibitory concentration (IC_50_) values of relacin (~840 µM; 46) and DMNP (~195–303 µM; 30), highlighting how difficult it would be to apply these compounds in the living organisms.

Another challenge is that (p)ppGpp synthetases are widespread and highly conserved among different bacterial species, including those that are part of the natural human microbiota. The use of drugs targeting these enzymes would thus be the same as using non-discriminatory antibiotics, although their ability to penetrate through the Gram(-) and Gram(+) cellular membranes might be a means of affording some specificity. An interesting alternative would be targeting the RSH enzymes regulatory domains instead of their catalytic centers. For example, it has been shown that regulatory domains of RSH enzymes encoded by the pathogenic strains of *P. aeruginosa*, *Klebsiella pneumoniae*, *Shigella flexneri*, and *Listeria monocytogenes* display greater interspecies differences than their highly conserved catalytic domains (47).

Also, an important question to consider is whether it is better to target (p)ppGpp synthesis (either through inhibition or induction) or hydrolysis. All of these approaches may bring beneficial results, however they each have their drawbacks as well. For instance, although (p)ppGpp synthesis inhibition may limit virulence gene expression, it is well known that *E. coli* cells devoid of (p)ppGpp often give rise to RNA polymerase mutants that in the absence of (p)ppGpp behave as if this alarmone was still there (48, 49); this would diminish the expected therapeutic effects in bacteria where RNA polymerase is (p)ppGpp’s direct target. In case of drugs inducing (p)ppGpp production, it can be imagined that if high cellular alarmone levels were induced, this would lead to growth inhibition; however, if these levels were not high enough, a possibly contrary effect would be obtained – virulence and pathogenicity pathways would be turned on and infection would proceed. Additionally, it has been shown recently that increased (p)ppGpp levels drive persister formation in bacteria, i.e. bacteria that are phenotypically resistant to antibiotics although genotypically they should be sensitive (50). Similar outcomes could be imagined for (p)ppGpp hydrolysis disruption, although if hydrolysis was to be fully inhibited, this approach might be the most promising since lack of an enzyme able to remove (p)ppGpp would be lethal (e.g. it is well known that *E. coli relA+ ΔspoT* mutants are nonviable (9)).

All in all, although targeting the stringent response to combat pathogens seems very attractive, many challenges remain. Nevertheless, continued research and a deeper understanding of this complex mechanism will be key to unlocking its full therapeutic potential.
